# Supplementary material for: An optical aptamer-based cytokine nanosensor detects macrophage activation by bacterial toxins
Source: bioRxiv. 2024 Apr 5:2024.04.05.588290. Preprint. [Version 1] doi: 10.1101/2024.04.05.588290 (PMC11014583; doi:10.1101/2024.04.05.588290)
Supplement: Supplement 1 [file media-1.pdf]

Supplementary Information for:

An optical aptamer-based cytokine nanosensor detects macrophage activation by bacterial toxins

Amelia K. Ryan[1], Syeda Rahman[1], Ryan M. Williams[1,2]*

[1]The City College of New York, Department of Biomedical Engineering, New York, NY 10031
[2]PhD Program in Chemistry, Graduate Center, City University of New York, New York, NY 10016
*correspondence to rwilliams4@ccny.cuny.edu

Supplementary Figures

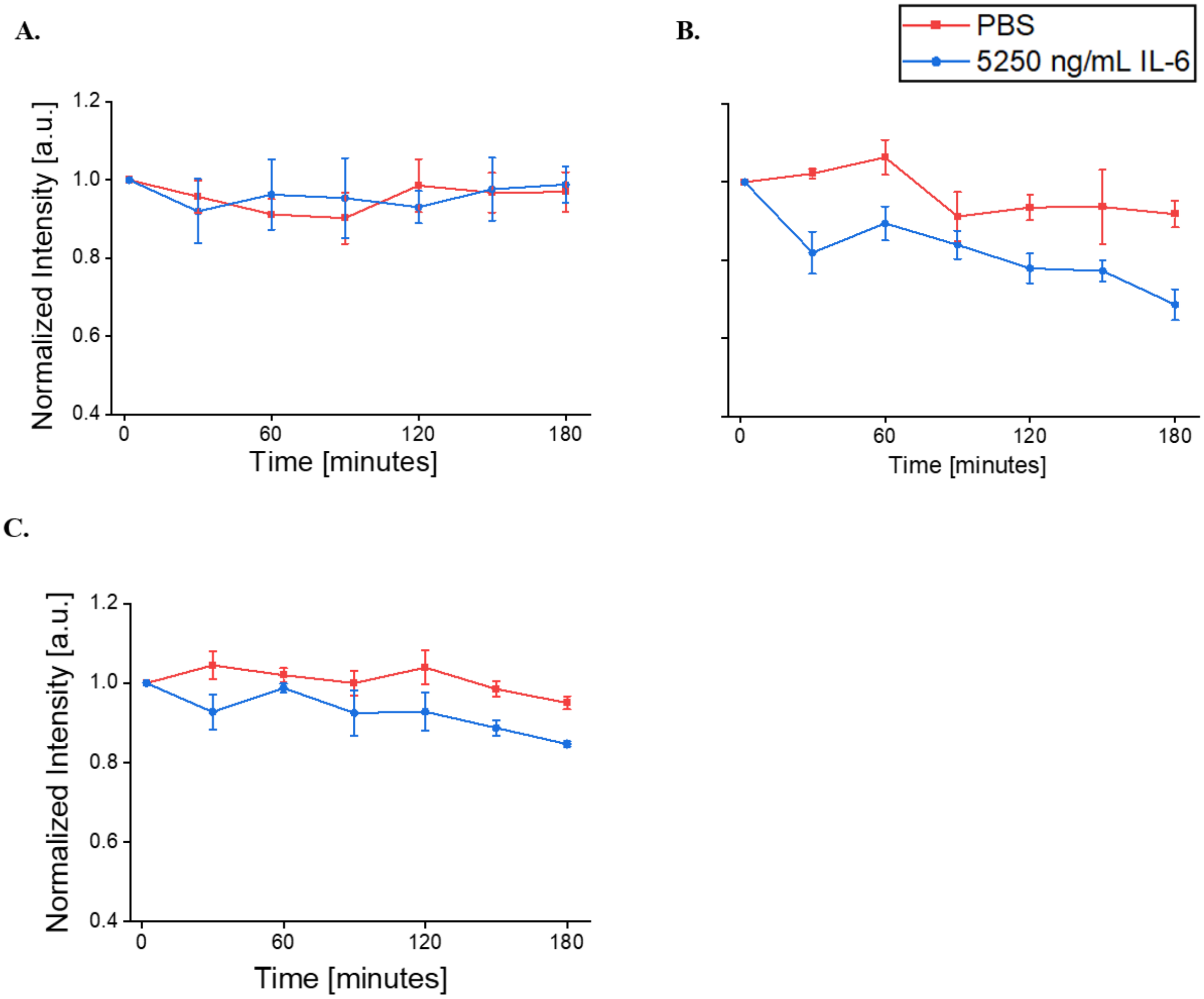

Figure S1. **Time-dependent response of SWCNT-ssDNA constructs to IL-6 in PBS.** A. (7,5) fluorescence intensity of SWCNT-(GT)$_{15}$ over three hours. B. (7,5) fluorescence intensity of SWCNT-(GT)$_{15}$+31Apt over three hours. C. (7,5) fluorescence intensity of SWCNT-15Apt over three hours

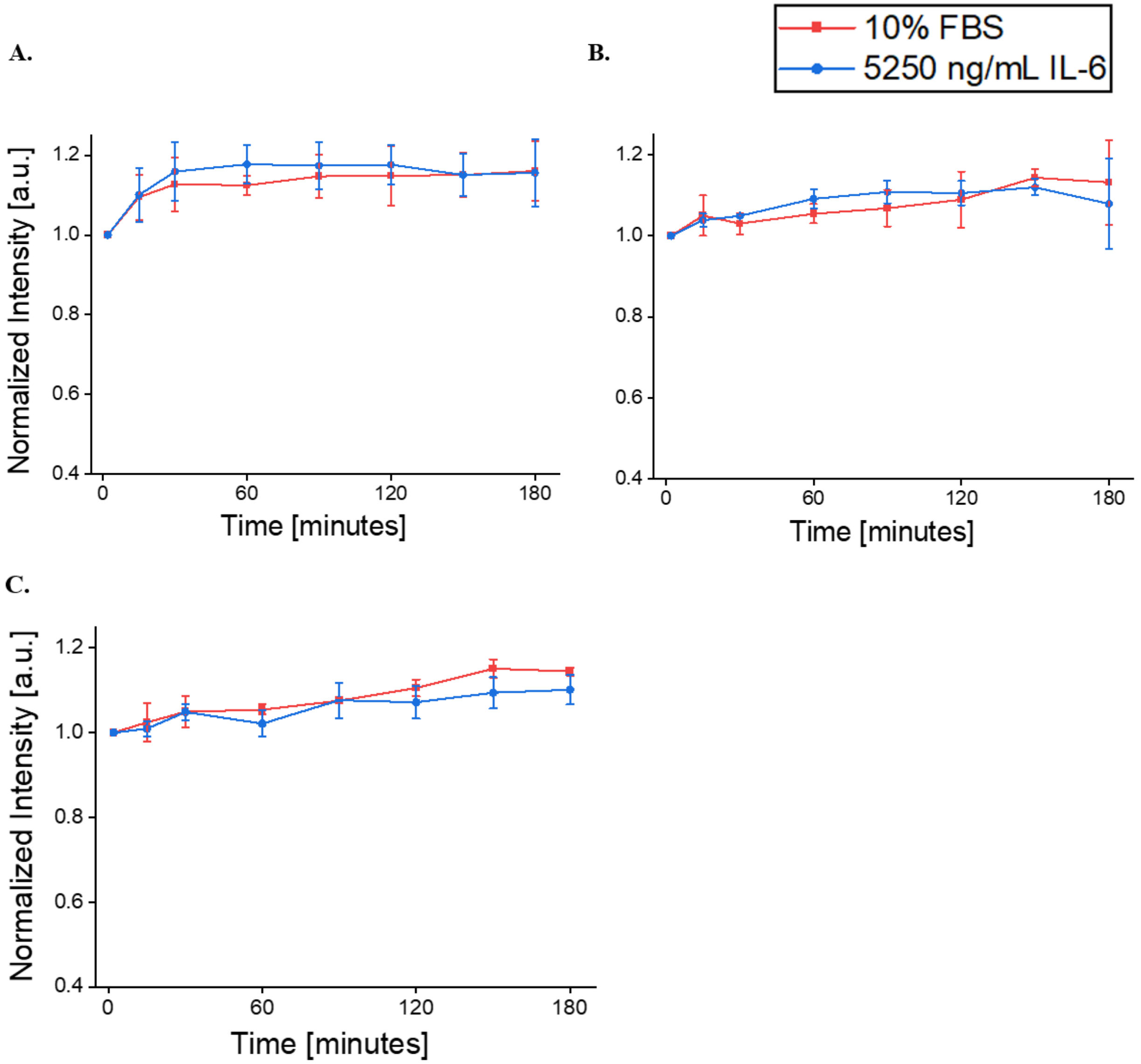

Figure S2. Time-dependent response of SWCNT-ssDNA constructs to IL-6 in PBS + 10% FBS. A. (7,5) fluorescence intensity of SWCNT-$(GT)_{15}$ over three hours. B. (7,5) fluorescence intensity of SWCNT-$(GT)_{15}$+31Apt over three hours. C. (7,5) fluorescence intensity of SWCNT-15Apt over three hours

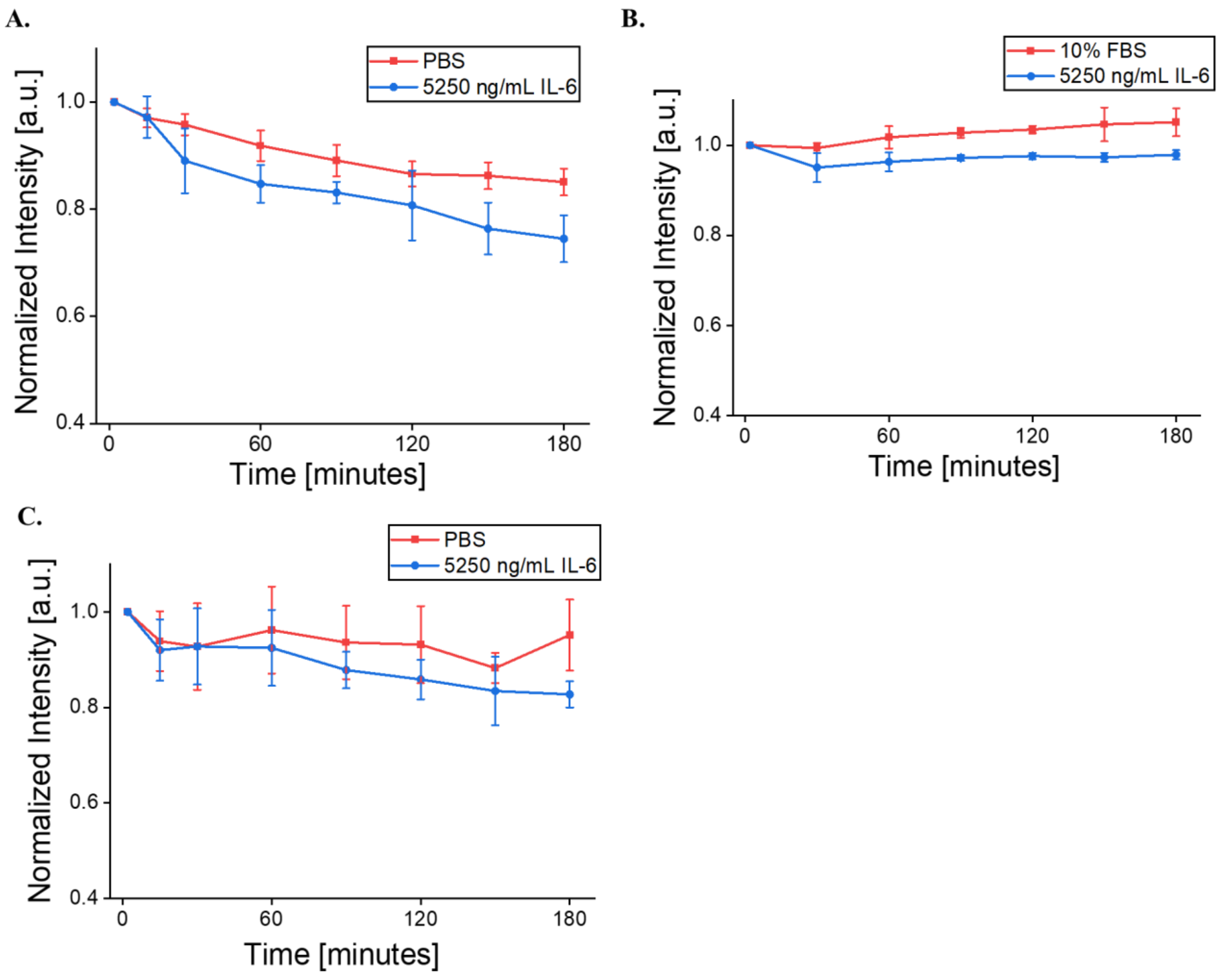

Figure S3. Mechanistic modulations of nanosensor. A. (7,5) Fluorescence intensity of heat-treated SWCNT-31Apt in response to IL-6 in PBS over three hours. B. (7,5) Fluorescence intensity of BSA-passivated SWCNT-31Apt in response to IL-6 in PBS + 10% FBS over three hours. C. (7,5) Fluorescence intensity of 0.02% SDBS-coated SWCNT-31Apt in response to IL-6 in PBS over three hours.

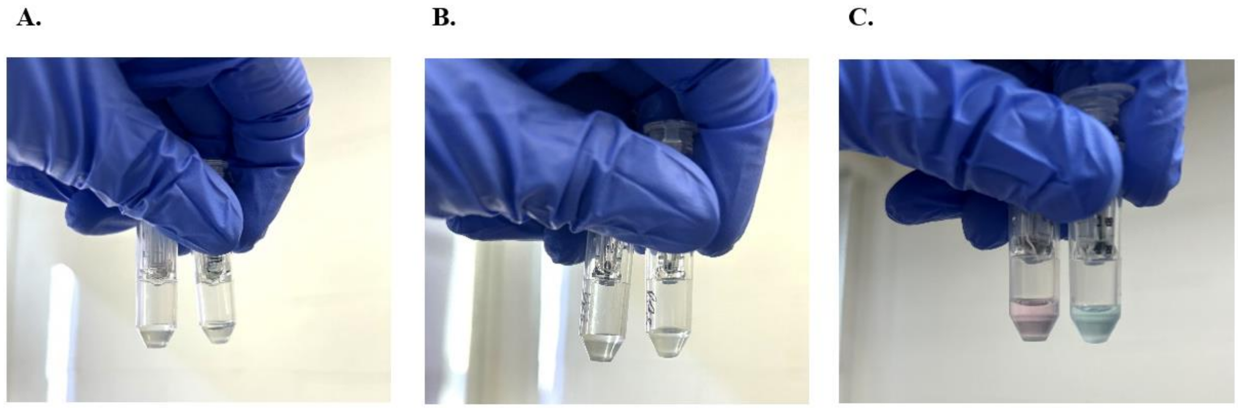

Figure S4. Photos of displaced cyanine dye-labeled DNA. A. Flow-through after incubation of SWCNT-$(GT)_{15}$+Cy3 (left) and SWCNT-31Apt+Cy5 (right) with PBS. B. Flow-through after incubation of SWCNT-$(GT)_{15}$+Cy3 (left) and SWCNT-31Apt+Cy5 (right) with 5250 ng/mL IL-6 protein in PBS. C. Flow-through after incubation of SWCNT-$(GT)_{15}$+Cy3 (left) and SWCNT-31Apt+Cy5 (right) with 2.5% DOC.

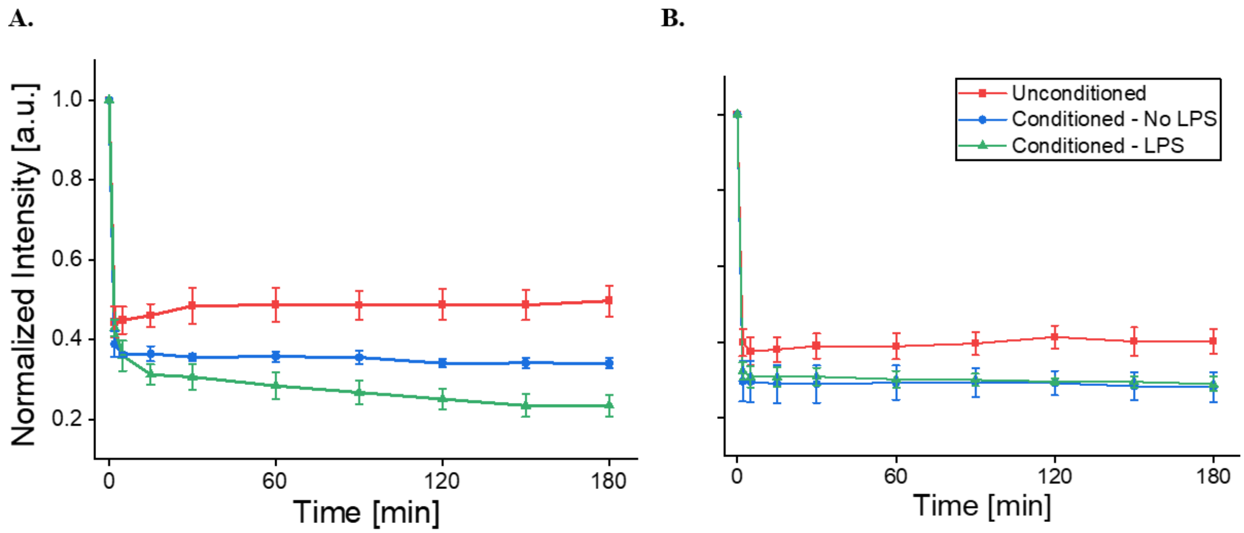

Figure S5. Time-sensitive response of SWCNT-ssDNA to conditioned media from Raw 264.7 cells. A) (7,5) Fluorescence intensity of SWCNT-31Apt in response to cell media samples over three hours. B) (7,5) Fluorescence intensity of SWCNT-$(GT)_{15}$ in response to cell media samples over three hours.
